# Letter to the editor: The role of angiotensin-converting enzyme inhibitors and angiotensin receptor blockers in developing a COVID-19 comorbidity-based host risk score

**DOI:** 10.1186/s13054-020-02903-9

**Published:** 2020-05-19

**Authors:** George D. Vavougios

**Affiliations:** 1 Department of Neurology, Athens Naval Hospital, 70 Deinokratous Street, 11125 Athens, Greece; 2 Department of Respiratory Medicine, University of Thessaly, Larisa, Greece; 3 Department of Computer and Electrical Engineering, Volos, Greece

To the Editor:

The recently published study by Shi et al. [1] described a risk score that provides a rapid assessment tool for COVID-19 patients. This score relies on comorbid hypertension to extract a cutoff point that identifies patients at risk of worse outcomes. While the idea is more than worthwhile in the clinical context, it has omitted a critical epidemiological and biological point regarding SARS-CoV-2: namely, ACE2 receptor hijacking. SARS-CoV-2, much like the structurally similar and precedent SARS-CoV, uses the ACE2 receptor to establish host uptake [2]. On the basis that ARBs and ACE inhibitors, commonly used medications in the treatment of hypertension, are known to increase ACE2 mRNA expression, Fang et al. [3] have hypothesized that COVID-19 patients with comorbid hypertension, treated with said antihypertensives, may be at an increased risk for developing the more severe forms of respiratory disease. Given previous knowledge on the SARS-CoV modus operandi, ACE2-dependent uptake was shown to determine de novo cytotoxicity to pancreatic islet cells and the development of hyperglycemia and diabetes [4].

On the premises of the article determining a host risk score, the lack of analyses for the use of ACE2 inhibitors among hypertensive patients detracts from the value of the developed score. Given that the effect of both ACE inhibitors/ARBs on disease course is largely unknown and the focus of further investigation [5], relevant treatment data would be invaluable both with respect to the score, and as within a broader epidemiological scope.

An analysis of this subgroup would not only increase the validity of the presented score, but also serve as clinical evidence on whether ACE2 inhibitors affect outcomes and COVID-19 phenotypes.

## Author’s response

### The uncertainty of ACEI/ARB on COVID-19 phenotypes

Yu Shi, Jifang Sheng

We appreciate the comment by Dr. George D. Vavougios on our recent paper and recognize the importance of clarifying the biological effect of either angiotensin-converting enzyme inhibitors (ACEI) or angiotensin II receptor blocker (ARB) on disease phenotypes of COVID-19 with the ongoing pandemic. There is the concern that the use of RAAS inhibitors may upregulate the level of ACE2 or alter its activity which serves as the functional receptor of SARS-CoV-2, thereby enhance viral infectivity and increase disease virulence. However, the data available so far do not support the hypothesis. First, the effects of RAAS inhibitors on ACE2 expression are uncertain. Experiments in animal models report conflicting findings in regard to the effects of ACE/ARB on the expression or activity of tissue ACE2 [6]. And very few data has been generated in human, especially regarding ACE/ARB on lung-specific expression of ACE2 [6]. Second, the causal relationship between ACE2 overexpression and viral infectivity or severe COVID-19 has not been established and other receptors or cofactors may be involved in viral infection process. Actually, the use of RAAS inhibitors may be beneficial in patients with COVID-19. It has been demonstrated in the experimental mouse model of SARS-CoV-1 that RAAS blockade alleviates lung injury, perhaps by reducing angiotensin II accumulation in the lung [7]. Additionally, ACE/ARB is well-recognized in promoting recovery from myocardial injury [8]. Furthermore, two recent studies provide preliminary evidence supporting the potential benefit of ACE/ARB in patients with COVID-19. One enrolled 476 patients both from Wuhan and outside Wuhan [9]. In line with our study, the incidence of hypertension was more frequent in critically illed patients than in the moderate group (35.7% vs 20.7%, *p* < 0.05). The study showed that ACEI/ARB inhibitors were less used in severe and critically illed groups than mild-moderate groups (6.1% vs 87.9%). Another enrolled a total of 417 cases, with 51 (12.23%) had hypertension [10]. Only 4 patients (23.5%) taking ACEI or ARB were severe, in contrast to 12 cases (48%) without the use of these agents. Therefore, current data favor a beneficial rather than pathogenic role of ACEI or ARB in COVID-19. If so, we actually underestimate the risk of hypertension in relation to the severe phenotype of COVID-19 when the use of ACEI or ARB is not taken into consideration. Nevertheless, we completely agree with the comment that data with respect to the use of RAAS inhibitors and illness severity of COVID-19 are needed, which should be generated by well-designed, high-quality, and large-scale randomized clinical trials or cohort studies enrolling subjects with multi-ethnicities.

## Data Availability

Not applicable.

## References

[1] Shi Y, Yu X, Zhao H, Wang H, Zhao R, Sheng J (2020). Host susceptibility to severe COVID-19 and establishment of a host risk score: findings of 487 cases outside Wuhan. Crit Care.

[2] Hoffmann Markus, Kleine-Weber Hannah, Schroeder Simon, Krüger Nadine, Herrler Tanja, Erichsen Sandra, Schiergens Tobias S., Herrler Georg, Wu Nai-Huei, Nitsche Andreas, Müller Marcel A., Drosten Christian, Pöhlmann Stefan (2020). SARS-CoV-2 Cell Entry Depends on ACE2 and TMPRSS2 and Is Blocked by a Clinically Proven Protease Inhibitor. Cell.

[3] Fang Lei, Karakiulakis George, Roth Michael (2020). Are patients with hypertension and diabetes mellitus at increased risk for COVID-19 infection?. The Lancet Respiratory Medicine.

[4] Yang JK, Lin SS, Ji XJ, Guo LM (2010). Binding of SARS coronavirus to its receptor damages islets and causes acute diabetes. Acta Diabetol.

[5] Patel AB, Verma A. COVID-19 and angiotensin-converting enzyme inhibitors and angiotensin receptor blockers: what is the evidence?. JAMA. 2020; [published online: March 24, 2020] doi: 10.1001/jama.2020.4812. 10.1001/jama.2020.4812 32208485

[6] Vaduganathan Muthiah, Vardeny Orly, Michel Thomas, McMurray John J.V., Pfeffer Marc A., Solomon Scott D. (2020). Renin–Angiotensin–Aldosterone System Inhibitors in Patients with Covid-19. New England Journal of Medicine.

[7] Kuba K, Imai Y, Rao S, Gao H, Guo F, Guan B, Huan Y, Yang P, Zhang Y, Deng W (2005). A crucial role of angiotensin converting enzyme 2 (ACE2) in SARS coronavirus-induced lung injury. Nat Med.

[8] Latini R, Tognoni G, Maggioni AP, Baigent C, Braunwald E, Chen ZM, Collins R, Flather M, Franzosi MG, Kjekshus J (2000). Clinical effects of early angiotensin-converting enzyme inhibitor treatment for acute myocardial infarction are similar in the presence and absence of aspirin: systematic overview of individual data from 96,712 randomized patients. Angiotensin-converting Enzyme Inhibitor Myocardial Infarction Collaborative Group. J Am Coll Cardiol.

[9] Feng Y, Ling Y, Bai T, Xie Y, Huang J, Li J, Xiong W, Yang D, Chen R, Lu F, et al. COVID-19 with different severity: a multi-center study of clinical features. Am J Respir Crit Care Med. 2020. 10.1164/rccm.202002-0445OC PMC7258639 32275452

[10] Meng J, Xiao G, Zhang J, He X, Ou M, Bi J, Yang R, Di W, Wang Z, Li Z (2020). Renin-angiotensin system inhibitors improve the clinical outcomes of COVID-19 patients with hypertension. Emerg Microbes Infect.

